# Propofol Infusion Syndrome in Adults: A Clinical Update

**DOI:** 10.1155/2015/260385

**Published:** 2015-04-12

**Authors:** Aibek E. Mirrakhimov, Prakruthi Voore, Oleksandr Halytskyy, Maliha Khan, Alaa M. Ali

**Affiliations:** Department of Internal Medicine, Saint Joseph Hospital, 2900 N. Lake Shore, Chicago, IL 60657, USA

## Abstract

Propofol infusion syndrome is a rare but extremely dangerous complication of propofol administration. Certain risk factors for the development of propofol infusion syndrome are described, such as appropriate propofol doses and durations of administration, carbohydrate depletion, severe illness, and concomitant administration of catecholamines and glucocorticosteroids. The pathophysiology of this condition includes impairment of mitochondrial beta-oxidation of fatty acids, disruption of the electron transport chain, and blockage of beta-adrenoreceptors and cardiac calcium channels. The disease commonly presents as an otherwise unexplained high anion gap metabolic acidosis, rhabdomyolysis, hyperkalemia, acute kidney injury, elevated liver enzymes, and cardiac dysfunction. Management of overt propofol infusion syndrome requires immediate discontinuation of propofol infusion and supportive management, including hemodialysis, hemodynamic support, and extracorporeal membrane oxygenation in refractory cases. However, we must emphasize that given the high mortality of propofol infusion syndrome, the best management is prevention. Clinicians should consider alternative sedative regimes to prolonged propofol infusions and remain within recommended maximal dose limits.

## 1. Introduction

Propofol is a sedative-hypnotic medication commonly used as an induction agent for preoperative sedation, prior to endotracheal intubation and other procedures as well as for sedation in the intensive care unit [1]. Its use was approved by the food and drug administration (FDA) in November 1989. Propofol administration has many important advantages, such as a rapid onset of action—within seconds after administration—and a short duration of action—up to 15 minutes [2]. Propofol possesses sedative, anxiolytic, and anticonvulsant properties [2]. Furthermore, propofol may have beneficial anti-inflammatory and antioxidative effects as well as neuroprotective properties including reduction of intracranial pressure [2]. Common side effects to anticipate after administration of propofol include a decrease in heart rate and in blood pressure [1].

The mechanism of action is not clear, but propofol seems to stimulate *γ*-aminobutyric acid receptors, block N-methyl-D-aspartate receptors, and diminish calcium influx via slow calcium ion channels [2].

Nevertheless, it has become obvious that propofol is not without risks. The first reported death associated with propofol infusion was of a 3-year-old girl in Denmark in 1990 [3]. This patient developed high anion gap metabolic acidosis (HAGMA), hypotension, and polyorgan failure [3]. In 1992 Parke et al. reported the deaths of five children who had similar presentations to the Danish case while being on propofol infusion [4]. The term “propofol infusion syndrome” (PRIS) first appeared in pediatric literature and was proposed by Bray who had reviewed 18 pediatric cases [5]. The clinical spectrum of PRIS consists of bradycardia, cardiovascular collapse, HAGMA, rhabdomyolysis, hepatomegaly, and lipemia [5].

Later, in 1996, the first adult case of lactic acidosis associated with propofol administration was reported [6]. The patient was a 30-year-old female who was admitted for bronchial asthma exacerbation and who had developed unexplained lactic acidosis [6]. Propofol infusion was stopped, and the lactic acidosis resolved with a favorable outcome [6]. Unfortunately, in 1998 a first adolescent mortality associated with propofol use was reported in a 17-year-old male with refractory status epilepticus [7].

The goal of this paper is to summarize the current knowledge on PRIS, primarily in adults (≥18 years old). First, we will review the pathogenesis of PRIS. Second, the epidemiology and proposed risk factors for PRIS will be discussed. Third, clinical presentation and diagnosis of PRIS will be reviewed. Finally, we will finish with the discussion of its prevention and the treatment for established PRIS.

## 2. Pathophysiology of Propofol Infusion Syndrome

Before reviewing the pathogenesis of PRIS, it is important to gain a basic understanding of energy metabolism. Under physiological circumstances, glucose is a major source of energy to the brain, the cardiac system, and skeletal muscles [8]. However, during stress conditions, there is a shift towards utilization of free fatty acids as a major source of energy for the vast majority of biological tissues. This shift in energy metabolism is achieved via the activation of stress hormones such as epinephrine and cortisol, which modulate the activity of hormone sensitive lipase in the adipose tissue. Hormone sensitive lipase in turn promotes the degradation of triglycerides into glycerol and free fatty acids. Both of these triglyceride constituents are taken by the liver cells: glycerol may be used as a source for glucose synthesis de novo, and free fatty acids are used in the mitochondrial beta-oxidation. This change in energy sources is quite important and aims to provide more glucose to the central nervous system and to the red blood cells. Beta-oxidation of fatty acids produces biochemical intermediates, which are used in the citric acid (also known as Krebs) cycle, which provide electrons to the electron transport chain and are used in the synthesis of ketone bodies, which can also be utilized as an energy source [8].

Because propofol is a hydrophobic substance, lipid emulsion is used as its solvent. A rabbit model showed both lipid solvent and propofol itself contribute to the development of hyperlipidemia and hypertriglyceridemia, which are commonly seen as features of PRIS [9]. However, the pathogenesis of PRIS is a very complex process and is not just a result of solvent lipid emulsion. Current understanding of PRIS includes the fact that it involves an intricate interplay between propofol-mediated biochemical changes that underlie the host state (e.g., sepsis, shock, cranial trauma, etc.) and the concomitant use of other pharmacological agents.

Propofol inhibits the activity of the carnitine palmitoyl transferase I, an outer membrane mitochondrial enzyme [10]. This enzyme transfers the fatty acyl group to carnitine to form fatty-acyl carnitine [8]. Fatty acyl carnitine can then be transported through the inner mitochondrial membrane where its metabolites participate in the citric acid cycle, ketone body production, and the electron transport chain [8]. Indeed, analyses of PRIS cases have shown accumulation of acylcarnitine in reported patients [11–13]. Due to propofol-mediated defects in beta-oxidation of fatty acids, fatty acids tend to accumulate in various organs (e.g., liver). Thus, patients with PRIS have elevated levels of FFA, which has actually been shown to promote cardiac arrhythmogenicity [14].

Furthermore, propofol is known to directly affect the mitochondrial electron transport chain. Animal studies have demonstrated that propofol uncouples oxidative phosphorylation [15], inactivates cytochrome c, and cytochrome a/a3 [16] as well as decreasing electron complex chain complex II, complex III, and coenzyme Q activity [17]. Clinical data have shown a decrease in cytochrome c oxidase activity [18] and electron transport chain complex IV activity [19].

Other factors that may contribute to the development of PRIS include decreased carbohydrate stores, advanced stress, and/or catecholamine administration and use of glucocorticoids. Again, we point out that PRIS was first recognized in the pediatric population [3, 5]. Carbohydrate depletion will lead to a reduction in citric acid levels, which slows lipid metabolism [8]. Animal models show that propofol inhibits beta-adrenergic receptors [20] thereby explaining why patients on propofol may require higher doses of exogenous catecholamine. On the other hand, an increase in catecholamines leads to greater clearance of propofol [21], which may, potentially, lead to the need of a higher propofol dose. Administration of glucocorticoids may potentiate protein degradation in both skeletal and cardiac muscle cells, which may contribute to cellular death [22]. Moreover, glucocorticosteroids and catecholamines are stress hormones that enhance lipolysis [8].

Furthermore, as was described above, propofol has calcium channel blocking properties on the heart, which lead to decreased cardiac performance [23] and promote inflammation in the cardiac muscle [24]. It is also possible that some patients who develop PRIS have a subclinical mitochondrial disorder [25, 26].

Thus, patients with PRIS have decreased energy availability at a time of increased demand (underlying critical illness, shock, etc.). This energy deprivation and imbalance might explain the observed myocytolysis of both skeletal and cardiac muscles in patients with PRIS [7, 26]. Muscle death leads to elevations in creatine kinase, myoglobin, potassium, and lactic acid. Rhabdomyolysis is a strong risk factor for acute kidney injury, which, if it occurs, may worsen metabolic acidosis. As was described above, propofol has numerous pathways to negatively affect the heart [14, 20, 23]. Furthermore, metabolic acidosis by itself creates an arrhythmogenic environment [27]. On the other hand, heart dysfunction may further worsen kidney function and metabolic acidosis due to cardiogenic shock. It is also important to note that features of the primary illness (sepsis, other forms of shock, status epilepticus, etc.) may overlap with PRIS and explain the features of PRIS in some of the cases [28, 29].

## 3. Epidemiology of the Propofol Infusion Syndrome

It is important to note that most of the clinical data on PRIS originates from case reports and case series. This paper's primary focus is on adult PRIS. Another problem when analyzing the epidemiological data on PRIS is the fact that different criteria were used for the diagnosis of PRIS. However, Hwang et al. estimated that PRIS incidence is around 17% in those receiving at least 5 mg/kg/hour and around 31% in those receiving more than 6 mg/kg/hour [29]. It is possible that the incidence of PRIS is even higher in patients who have other risk factors for the development of PRIS (such as shock, use of catecholamines, use of glucocorticosteroids, and carbohydrate depletion). The risk factors for PRIS will be discussed in greater detail below. A summary of the 37 total reported adult PRIS cases is presented in Table 1 [25, 26, 30–55]. Of these 37, unfortunately only 7 patients survived [41, 45, 47, 50–52].

In a prospective multicenter study involving 11 academic medical centers, patients who were receiving propofol infusion for at least 24 hours were monitored for the development of PRIS [56]. PRIS was defined as metabolic acidosis plus cardiac dysfunction and at least one other variable including the fact that hypertriglyceridemia, rhabdomyolysis, and acute kidney injury occurred after the start of propofol. PRIS was found among 1.1% of patients (11 patients out of 1,017). We found that people who developed PRIS had been receiving propofol on average for three days. Eighteen percent of the patients with PRIS received a propofol dose for more than 83 mcg/kg/minute, and 18% of the patients died. Also, patients who developed PRIS had higher APACHE II scores.

In conclusion, further prospective studies are needed to delineate better whether certain factors play a role in PRIS occurrence (age, gender, underlying medical condition, etc.), its incidence, natural history, and management.

## 4. Risk Factors for the Development of Propofol Infusion Syndrome and Its Prevention

When assessing the potential risk factors for the development of PRIS among adults, one must keep in mind that some of the data that apply actually came from pediatric research studies. For example, the notion that low carbohydrate stores play a role in the pathogenesis of PRIS came from a pediatric study [11]. Thus, the possibilities of generalizing the findings and applying them to the adult population are unclear.

Nevertheless, certain risk factors or risk markers for the development of PRIS merit discussion. First of all, based on its name, PRIS cannot develop without current or recent propofol administration. As was discussed above, propofol is a popular choice for sedation in the ICU setting. However, PRIS occurs predominantly in patients receiving high doses for a prolonged period (see Table 1). As was shown by Cremer et al., the odds for PRIS increase significantly with higher propofol doses [30]. Thus, based on the data from case reports and case series, administering propofol for more than 48 hours is not recommended, nor is it to administer a dose of more than 4 mg/kg/hour or 67 mcg/kg/minute.

Other potential risk factors for the development of PRIS are critical illness (sepsis, head trauma, status epilepticus, etc.), use of vasopressors and glucocorticosteroids, carbohydrate depletion (liver disease, starvation, or malnutrition), carnitine deficiency, and subclinical mitochondrial disease [28, 57–59]. It is not clear whether these factors represent only a marker of a severe illness or if they play a direct role in the development of PRIS. Furthermore, subclinical mitochondrial disease is a risk marker for PRIS that was only reported in pediatric literature. However, supplementary carbohydrate administration at 6–8 mg/kg/minute might, possibly, mitigate the risk of PRIS [22].

Thus based on the factors above, clinicians must keep a high index of suspicion for the development of PRIS. The duration of propofol administration should not exceed 48 hours, and the dose should not be higher than 4 mg/kg/hour nor greater than 67 mcg/kg/minute. Schroeppel et al. demonstrated that daily serum creatine kinase (CK) measurements to detect increased levels while on propofol may detect a high risk group for the development of PRIS [60]. In particular, they used a cut-off of less than 5,000 U/L to represent a low-risk population for the development of PRIS. Indeed, this study has shown that the incidence of PRIS was only 0.19% in patients deemed to be low risk for PRIS. Nevertheless, future studies are needed to replicate this approach and potentially find new biomarkers for an early detection of PRIS risk.

In conclusion, clinicians must be aware of the potential for PRIS in patients receiving propofol and restrict the duration and the dose of propofol to the limits described above. It is unclear whether carbohydrate supplementation, avoidance of vasopressors (particularly catecholamines), and glucocorticosteroids (whenever possible) will translate into a reduced risk of PRIS. However, whenever feasible, avoiding these medications (glucocorticosteroids and catecholamines) is advised for patients receiving propofol.

## 5. Clinical Presentation and Diagnosis of Propofol Infusion Syndrome

As was discussed above, the pathogenesis of PRIS involves the interaction between enhanced lipolysis, impaired fatty acid oxidation, mitochondrial dysfunction, underlying critical illness, and concurrent medication use (like catecholamines and glucocorticosteroids).

Common organ systems affected by PRIS include the cardiovascular, the hepatic, the skeletal muscular, the renal, and the metabolic. Cardiovascular manifestations of PRIS include widening of QRS complex, Brugada syndrome-like patterns (particularly type 1), ventricular tachyarrhythmias, cardiogenic shock, and asystole. Skeletal muscle manifestations include myopathy and overt rhabdomyolysis. Skeletal muscle injury may be complicated by hyperkalemia and acute kidney injury. Metabolic manifestations of PRIS also include HAGMA (due to elevation in lactic acid). However, other causes of elevated lactic acid, such as other forms of shock (septic, cardiogenic, etc.), tissue ischemia (bowel, limb), and certain medications (epinephrine, beta 2 agonists, etc.) may account for elevated lactic acid [61]. Metabolic acidosis can further worsen hyperkalemia due to increased transcellular shift [62]. Hepatic manifestations include liver enzymes elevation, hepatomegaly, and steatosis. Hypertriglyceridemia is an expected side effect of propofol administration, and it is unclear whether this alone represents a true feature of PRIS. It is prudent to emphasize that PRIS lacks specific signs and symptoms (other than propofol administration) and its presentation overlap greatly with other conditions leading to critical illness (various forms of shock, renal disease due to other causes, etc.). Therefore, clinicians should keep a broad differential in mind while managing a patient with possible PRIS.

On the other hand, PRIS must be considered if suggestive clinical features (e.g., HAGMA or cardiac arrhythmias) develop in patients receiving high dose (>4 mg/kg/hour or >67 mcg/kg/minute) and/or prolonged infusions of propofol (≥48 hours). Clinicians may consider screening patients for PRIS with CK measurements that have been shown to detect patients at high risk for the development of PRIS [60].

## 6. Treatment of Established Propofol Infusion Syndrome

As was discussed above, clinicians should keep a high index of suspicion for PRIS. Of a particular note, new onset and otherwise unexplained HAGMA, cardiac dysfunction (Brady or tachyarrhythmias, Brugada syndrome-like patterns on ECG, and cardiogenic shock and asystole), elevated liver and pancreatic enzymes, hypertriglyceridemia, rhabdomyolysis, hyperkalemia, and acute kidney injury should warrant strong consideration of PRIS. The notion that prevention of a disease is always better than the treatment of an established disease is very true for PRIS, given the high associated mortality rate. Therefore, clinicians should aim to limit the duration of propofol use (not more than 48 hours) and dosage (not more than 4 mg/kg/hour or 67 mcg/kg/minute). Substitution with a different sedative agent should be considered once the patient reaches the aforementioned limits.

As was described above and presented in Table 1, all of our knowledge on the management of PRIS is based on case reports and case series. Unfortunately, most patients with reported PRIS have died. Of note, there is no specific antidote or treatment targeted against PRIS. The management approach in described cases is purely supportive and targeted to the features of PRIS.

First line therapy of suspected PRIS is to immediately discontinue the administration of propofol. Management of metabolic acidosis in the reported cases includes administration of sodium bicarbonate and renal replacement therapy. However, the role of sodium bicarbonate in the management of lactic acidosis is quite controversial and not universally accepted [63, 64]. Hyperkalemia and rhabdomyolysis are strong indications to consider renal replacement therapy for patients with metabolic acidosis due to PRIS. Also, patients with hyperkalemia and rhabdomyolysis should receive vigorous fluid administration [65]. However, euvolemia should be maintained in patients with traumatic brain injuries, which is a common comorbid condition in patients who have developed PRIS [66]. Calcium administration (either chloride or gluconate), insulin with or without dextrose, b2 agonist administration, sodium bicarbonate, and potassium binding resin can also be considered in the management of hyperkalemia [67].

Cardiac dysfunction and arrhythmias represent a major cause of mortality in patients with PRIS. Bradyarrhythmias were managed with transvenous pacing in the reported cases. Aggressively managing the hyperkalemia is important, given the fact that it can detrimentally affect cardiac function. Appearance of Brugada-like patterns on the ECG should be considered as anonymous sign, which may represent an increased risk of ventricular tachyarrhythmias. Cardiac arrest should be managed according to the American Heart Association Advanced Cardiovascular Life Support guidelines [68]. Cardiogenic shock should be managed with the support of vasopressors and inotropes, such as norepinephrine and dobutamine, for example, and mechanical devices in refractory cases [69, 70]. However, propofol pharmacology includes the blockage of cardiac calcium channels and beta blocking properties, thus making the use of catecholamine mimetic potentially less efficacious [20, 23]. Based on theoretical data that the inhibition of phosphodiesterase via medications (such as milrinone, e.g.), the administration of glucagon, and calcium may bypass the effect of propofol on these receptors, some advocate the use of the aforementioned agents for augmenting cardiovascular support [20, 23]. In refractory cases of PRIS, extracorporeal membrane oxygenation should be strongly considered [52]. It is important to mention that managing other aspects of regular intensive unit care is important—such as the prevention of ventilator-associated pneumonia and other infections, deep venous thrombosis prophylaxis, stress ulcer prophylaxis, decubitus ulcer prophylaxis, and skin care as well as nutritional support. Of particular importance, carbohydrate administration may prevent or mitigate the risk of the development of PRIS [11, 28, 71]. It is unclear whether carnitine supplementation will result in decreased risk of PRIS.

In conclusion, the best management of PRIS lies in its prevention. Complications of PRIS such as hyperkalemia, acute renal failure, cardiovascular collapse, and malignant arrhythmias should be aggressively treated.

## 7. Conclusion

PRIS is a rare but extremely dangerous complication of propofol administration with a high mortality. Certain risk factors for the development of PRIS are described, such as inappropriate propofol doses and durations of administration, carbohydrate depletion, severe illness, and concomitant administration of catecholamines and glucocorticosteroids. The pathophysiology of this condition includes impairment of mitochondrial beta-oxidation of fatty acids, disruption of the electron transport chain and blockage of beta adrenoreceptors, and cardiac calcium channels. The disease commonly presents as an otherwise unexplained HAGMA, rhabdomyolysis, hyperkalemia, acute kidney injury, elevated liver enzymes, and cardiac dysfunction. Management of overt PRIS includes immediate discontinuation of propofol infusion and problem-driven management, including hemodialysis, hemodynamic support, and extracorporeal membrane oxygenation in refractory cases. However, we must emphasize that, given the high mortality of PRIS, the best management is prevention. Clinicians should consider alternative sedation agents in patients who are receiving prolonged or high-dose propofol infusions.

## Figures and Tables

**Table 1:** Summary of reported PRIS cases in adults.

Authors [ref.]	Year and country	Age and gender	Underlying pathology	Propofol dose and duration	PRIS features	Treatment and outcome
Stelow et al. [31].	2000; USA	47-year-old female and 41-year-old male	Bronchial asthma exacerbation	200–222 mcg/kg/minute and >48 hours	Rhabdomyolysis, hyperkalemia, cardiovascular collapse (female). Both patients were also treated with glucocorticosteroids for asthma	Renal replacement therapy, vasopressors. Female patient died, the outcome for a male patient not reported.

Perrier et al. [32].	2000; USA	18-year-old male	Multiple trauma (including closed head trauma) after motor vehicle accident	≥50 mg//hour and 98 hours	Bradycardia, left bundle branch block, lactic acidosis, rhabdomyolysis, and hyperkalemia and cardiovascular collapse (pulseless electrical activity and asystole)	Inotropes, atropine. The patient died.

Cremer et al. [30].	2001; Netherlands	7 patients aged 16–55 years (no specific data provided)	Acute traumatic brain injury	5.5 mg/kg/hour–7.4 mg/kg/hour; 65–177 hours	Cardiac arrhythmias in all patients, metabolic acidosis in 6 patients hyperkalemia in 6 patients, rhabdomyolysis in 4 patients, and lipemia in 3 patients	Pressors and inotropes. All patients died.

Badr et al. [33].	2001; USA	21-year-old female	Spontaneous intracerebral hemorrhage due to arteriovenous malformation	4.5–9 mg/kg/hour; >48 hours	Metabolic acidosis, cardiovascular collapse	Pressors, inotropes, intravenous bicarbonate. The patient died.

Friedman et al. [34].	2002; USA	23-year-old female	Status epilepticus	200 mcg/kg/minute; 106 hours	Metabolic acidosis, hyperkalemia, acute kidney injury, wide complex tachycardia, and cardiovascular collapse	The patient died, no treatment/management was reported.

Ernest and French [35].	2003; Australia	31-year-old male	Closed head injury	4 mg/kg/hour; 157 hours	Metabolic acidosis, acute kidney injury, rhabdomyolysis, and cardiovascular collapse	None reported. The patient died.

Casserly et al. [36].	2004; USA	42-year-old male	Cerebral venous thrombosis	12 mg/kg/hour; >96 hours	Metabolic acidosis, rhabdomyolysis, acute kidney injury, and cardiovascular collapse	Pressors, intravenous bicarbonate. The patient died.

Kumar et al. [37].	2005; USA	24-year-old female, 27-year-old female and 64-year-old male	24-year-old female with status epilepticus due to encephalitis, 27-year-old female with seizures due to intracerebral bleeding secondary to arteriovenous malformation and 64-year-old male with status epilepticus	2.6 mg/kg/hour for 64 year old male (non reported for others); 24–86 hours	Metabolic acidosis, hyperkalemia, rhabdomyolysis, acute kidney injury, and cardiovascular collapse	Inotropes, transvenous pacing, intravenous bicarbonate, intravenous calcium. All patients died.

Machata et al. [38].	2005; Austria	40-year-old male	Motor vehicle accident and cervical fracture	Dose not reported; 72 hours	Metabolic acidosis, hyperkalemia, acute kidney injury, and fever	Continuous venovenous hemofiltration. The patient died from septic complication.

Eriksen and Povey [39].	2006; Denmark	20-year-old female	Polytrauma	1.4–5.1 mg/kg/hour; 88 hours	Rhabdomyolysis, hyperkalemia, acute kidney injury, and cardiovascular collapse	Pressors, inotropes, intravenous bicarbonate. The patient died.

Merz et al. [40].	2006; Switzerland	24-year-old male	Cervical spine injury and acute respiratory distress syndrome. The patient received high dose methylprednisolone	2.6 mg/kg/hour (highest reported range); 86 hours	Hyperkalemia, rhabdomyolysis, acute kidney injury, and cardiovascular collapse	Pressors, inotropes. The patient died.

Corbett et al. [41].	2006; USA	21-year-old male	Traumatic brain injury	31.6–105.5 mcg/kg/minute; 3 days	Metabolic acidosis, rhabdomyolysis, and cardiac dysfunction	Supportive treatment. The patient survived.

Zarovnaya et al. [42].	2007; USA	31-year-old female	Status epilepticus	4.2–7.2 mg/kg/hour; 45 hours	Hyperkalemia, rhabdomyolysis, and cardiovascular collapse	Pressors, inotropes, transvenous pacing, renal replacement therapy. The patient died.

Orsini et al. [43].	2009; USA	36-year-old female	HIV, Pneumonia, and sepsis	1.5 mg/kg/hour; 7 days	Morbilliform rash, elevated liver enzymes, elevated pancreatic enzymes, elevated triglycerides, and hepatomegaly with hepatic fatty infiltration. The patient was also on glucocorticosteroids and vasopressors	Discontinuation of propofol infusion. The patient survived.

Ramaiah et al. [44].	2011; USA	42-year-old morbidly obese female	Elective parathyroidectomy	4 mg/kg/hour; 65 hours	Rhabdomyolysis, acute kidney injury, metabolic acidosis (also the patient developed septic shock secondary to ventilator associated pneumonia and urinary tract infection)	Vasopressors, renal replacement therapy. The patient survived her illness, but later died (65 days later, from tracheostomy occlusion in prone position due to fall).

Lee et al. [45].	2011; Korea	29-year-old female	Dilation and curettage for intrauterine fetal death	100 mg bolus dose	Hyperkalemia, metabolic acidosis, and cardiovascular arrest	Calcium gluconate, furosemide, inotropes. The authors deemed other potential causes like anaphylaxis, primary respiratory failure and amniotic fluid embolism to be unlikely in her case. The patient died.

Faulkner et al. [46].	2011; USA	23-year-old male	Traumatic brain injury and status epilepticus	4.8 mg/kg/hour; 5 days	Type I pattern of Brugada pattern on electrocardiography (ECG), rhabdomyolysis, hyperkalemia, hypertriglyceridemia, and metabolic acidosis	Intravenous hydration, plasma exchange. ECG findings resolved 48 hours after discontinuation of propofol. The patient survived.

Annecke et al. [47].	2012; Germany	36-year-old female	Severe head trauma	2.8 mg/kg/hour; 5 days	Rhabdomyolysis, Brugada syndrome pattern on ECG, hyperkalemia, metabolic acidosis, and cardiovascular collapse	Vasopressors, inotropes, hemofiltration, transvenous pacing. The patient died.

Mijzen et al. [48].	2012; Netherlands	23-year-old male	Open skull fracture	4.7–5.8 mg/kg/hour; 6 days	ECG changes (biphasic T waves, Brugada syndrome type 1 like pattern, S T segment depression, wide QRS complexes), hyperkalemia, metabolic acidosis, and cardiovascular collapse	Calcium gluconate, insulin and dextrose, hemodialysis. The patient died.

Vanlander et al. [26].	2012; Belgium	40-year-old male	Head trauma, underlying blindness	2.67–5.35 mg/kg/hour; 88 hours	Metabolic acidosis, rhabdomyolysis, Brugada syndrome type 1 like pattern. The patient was also on vasopressor	Carnithine, thiamine, vitamin B 12, renal replacement therapy. The patient died. Genetic testing demonstrated the presence of Leber hereditary optic neuropathy.

Deters et al. [49].	2013; USA	35-year-old male	Status epilepticus	150 mcg/kg/minute; 3 days	Rhabdomyolysis (day 3), metabolic acidosis, hyperkalemia, acute kidney injury, elevated liver enzymes, and Brugada syndrome like pattern (type 1)	Hemodialysis. The patient survived.

Agrawal et al. [50].	2013; India	53-year-old female	Polytrauma (subarachnoid hemorrhage, hepatic and pelvic bleeding, femoral neck fracture, and pelvic fractures)	20–65 mcg/kg/min; 5 days	Metabolic acidosis, hyperkalemia, and cardiovascular collapse	Vasopressors. The patient died.

Pothineni et al. [51]	2015; USA	25-year-old male	Head trauma and subdural hematoma	75–100 mcg/kg/minute; 3 days	Hyperkalemia, metabolic acidosis, rhabdomyolysis, acute kidney injury, elevated liver enzymes, and cardiovascular collapse	Amiodarone, lidocaine, continuous renal replacement therapy. The patient died.

Savard et al. [25].	2013; Canada	23-year-old female	Status epilepticus	10.7 mg/kg/hour; 69 hours	Metabolic acidosis and rhabdomyolysis. The patient was found to be positive for mutated polymerase gamma 1 mutation	Hemofiltration. The patient survived P RIS, but the care was later withdrawn (day 75) due to refractory status epilepticus and poor prognosis.

Mayette et al. [52].	2013; USA	20-year-old female	Status epilepticus	9 mg/kg/hour; 2 days	Shock, elevated liver enzymes, rhabdomyolysis, hyperkalemia, acute kidney injury, wide QRS, and ventricular tachycardia	Intravenous hydration, pressors, renal replacement therapy, extracorporeal membrane oxygenation. The patient survived.

Linko et al. [53].	2014; Finland	19-year-old female	Burn	Up to 6.95 mg/kg/hour; 11 days	Rhabdomyolysis, acute kidney injury, right-sided cardiac failure, and Brugada syndrome type 1 like pattern	Intravenous bicarbonate, continuous venovenous hemofiltration. The patient survived.

Bowdle et al. [54].	2014; USA	39 year old female	Vestibular schwannoma	Up to 160 mcg/kg/minute;	Hypertriglyceridemia (intraoperatively), elevated liver enzymes	The patient survived.

Diaz et al. [55].	2014; USA	38-year-old male	Abdominal gunshot wound	Up to 125 mcg/kg/minute; 5 days	Metabolic acidosis, rhabdomyolysis, hyperkalemia, acute kidney injury, hypertriglyceridemia, and elevated liver enzymes	Pressors, hemodialysis. The patient died.
